# Changes in the content of pollen total lipid and TAG in *Arabidopsis thaliana DGAT1* mutant *as11*

**DOI:** 10.1093/aobpla/plad012

**Published:** 2023-03-22

**Authors:** Mei Bai, Han Gao, Yang Yang, Hong Wu

**Affiliations:** State Key Laboratory for Conservation and Utilization of Subtropical Agro-bioresources, College of Life Sciences, South China Agricultural University, Guangzhou 510642, China; Guangdong Key Laboratory for Innovative Development and Utilization of Forest Plant Germplasm, College of Forestry, South China Agricultural University, Guangzhou 510642, China; State Key Laboratory for Conservation and Utilization of Subtropical Agro-bioresources, College of Life Sciences, South China Agricultural University, Guangzhou 510642, China; State Key Laboratory for Conservation and Utilization of Subtropical Agro-bioresources, College of Life Sciences, South China Agricultural University, Guangzhou 510642, China; State Key Laboratory for Conservation and Utilization of Subtropical Agro-bioresources, College of Life Sciences, South China Agricultural University, Guangzhou 510642, China; Guangdong Key Laboratory for Innovative Development and Utilization of Forest Plant Germplasm, College of Forestry, South China Agricultural University, Guangzhou 510642, China

**Keywords:** *Arabidopsis thaliana*, pollen development, TAG, tapetum

## Abstract

In mature pollen grains, lipids are primarily stored in the form of lipid droplets that provide energy and act as a carbon source for normal pollen development and germination. Triacylglycerol (TAG) is the major form of stored plant lipids. Diacylglycerol transferase, which is encoded by *DGAT1* in *Arabidopsis thaliana*, is an important enzyme regulating triacylglycerol synthesis. Within the seeds of the *DGAT1* mutant *as11*, the content of TAG is significantly decreased and the fatty acid composition also differs from the wild type. Transcriptome data of mature anthers showed that the genes involved in the TAG synthesis pathway were downregulated in *as11*. Analysis of gene expression patterns via transcriptome data also revealed that the expression of *PDAT1*, which functions in a manner complementary to the *DGAT1* gene, was significantly decreased in *as11*, whereas the amylopectin synthase genes *SS1* and *SS2* were upregulated in mutant *as11*. We also detected lower total lipid, TAG and fatty acid contents in mature *as11* pollen, with palmitic acid (C16:0) and linolenic acid (C18:3) being the major fatty acids in mature pollen. The cytological results showed that the lipid droplet content was reduced in mature *as11* pollen. In the binuclear pollen grain II stage, WT pollen contained lipid droplets that were primarily accumulated around the generative nucleus, whereas the pollen in the mutant *as11* was rich in starch grains that were primarily distributed around the vegetative nucleus. Ultrastructural analysis indicated that during pollen development in *as11*, the amount of endoplasmic reticulum in tapetal cells and pollen grains decreased, whereas the Golgi body content increased, which directly or indirectly led to a decrease in the levels of lipidosomes and an increase in the starch content in *as11*. Changes in the lipid content and fatty acid composition of the mutant as11 differ from those in the wild type during pollen development.

## Teaser

Diacylglycerol transferase, which is encoded by DGAT1 in *Arabidopsis thaliana*, is an important enzyme that regulates triacylglycerol synthesis. Transcriptome data of mature anthers showed that the genes involved in the TAG synthesis pathway were downregulated in DGAT1 mutant as11. The total lipid, TAG and fatty acid contents were reduced in as11 mature pollen, with palmitic acid (C16:0) and linolenic acid (C18:3) being the major fatty acids in mature pollen. The cytological results showed that the lipid droplet content was lower in mature as11 pollen compared to the wild type.

## Introduction

Pollen development in higher plants is a complicated process that involves the synthesis and secretion of nutriments such as carbohydrates, lipids, and others to provide nutrition for subsequent pollen development (Liu *et al*., 2011). Tapetal cells can also synthesize and secrete different nutriments at various stages throughout development to allow for the nourishment of pollen grains (Zhu *et al*., 2011; Chen *et al*., 2013). Typically, a large number of nutriments are accumulated within microspores at the binuclear pollen grain stage, and these nutriments are primarily lipids, storage proteins, and/or carbohydrates (Calzoni *et al*., 1990). For example, during pollen development in *Lilium*, a large number of starch grains are present within the vegetative cells at the early binuclear pollen grain stage. However, they begin to store lipid droplets as the primary nutriment in mature pollen (Clément *et al*., 1994). Interestingly, during pollen development in tomato and Asian lotus, the opposite phenomenon is observed, with more starch grains than lipid droplets in mature pollen (Zhu and Tian, 2015; Zhang *et al*., 2019). However, during pollen development in the Hong Kong orchid tree, lipid droplets are present at the early stage, the lipid droplet and starch grain contents increase at the late binuclear pollen stage, and mature pollen grains store both lipid droplets and starch grains, but with the lipid content being dominant (Zheng *et al*., 2017). At the binuclear pollen grain stage in Chinese cabbage, lipid droplets are abundant in both the tapetum and the pollen (Xie *et al*., 2005). Additionally, the transport and transformation of nutriments are both directly related to pollen fertility. The innermost layer of anther parietal cells (the tapetum) will select, transform and transport nutriments entering the anther locule. Therefore, the abnormal accumulation of nutriments within the pollen and tapetum may result in pollen abortion (Xie *et al*., 2005; Xu *et al*., 2009; Zhang *et al*., 2009; Yang *et al*., 2017).

Lipids and lipid derivatives are important components of pollen grains, particularly the mature pollen of most entomophilous flowers, and the lipid content can reach up to 25% of their dry weight (Murphy, 2001). Lipids that are accumulated in mature pollen can be divided into two categories. In the first category, the main components are phenolic substances and lipids. These are primarily composed of fatty acids and carotenoids that are synthesized and secreted by the tapetum and released into the anther locule to cover and deposit onto the extine to form the tryphine (Ischebeck, 2016). The second category includes intracellular self-synthesized lipids that are formed during pollen development and is primarily comprised of polar lipids that are related to membrane synthesis and neutral lipids that are stored in lipid droplets (Rodriguez-García *et al*., 2003; Ischebeck, 2016). Lipid droplets in mature pollen possess one unique and relatively simple structure and are similar to oil bodies that are stored in plant seeds. Triacylglycerol (TAG) and sterol ester are the main components, and the outside is covered by a phospholipid monolayer membrane that is inlaid with a variety of proteins (Thiam *et al*., 2013; Ohsaki *et al*., 2014). There are two sites in plants where lipid droplets are synthesized (chloroplasts and endoplasmic reticulum), and lipid droplets in mature pollen are primarily synthesized by the endoplasmic reticulum (Pyc *et al*., 2017). During late pollen development, lipid droplets are surrounded by a large number of endoplasmic reticulum structures to ensure that the lipid droplets are prevented from fusing with each other during pollen rehydration without the protection of oleosin (Murphy, 2001). TAG is stored in large amounts within lipid droplets and acts as the primary source for providing energy and as an inert intermediate that regulates lipid synthesis (Ziekiewicz *et al*., 2013). Therefore, TAG synthesis is essential for pollen development (Zhang *et al*., 2009; Ischebeck, 2016).

In addition to its content within pollen, TAG exists extensively in plant seeds and fruits as the most primary storage form of plant oil (Lu *et al*., 2003; Saha *et al*., 2006), where it provides a carbon source and the nutriments necessary for seed germination and development (Routaboul *et al*., 1999; Zou *et al*., 1999; Baud *et al*., 2002; O’Neill *et al*., 2003; Zheng *et al*., 2003; Lung and Weselake, 2006). Diacylglycerol transacylase (DGAT) is encoded by the *DGAT1* gene in *Arabidopsis thaliana* and is an important enzyme that catalyzes the synthesis of TAG. Compared to the wild type (WT), seeds from the *DGAT1* gene mutant *as11* exhibit a clear delay in development (Katavic *et al*., 1995; Hobbs *et al*., 1999; Routaboul *et al*., 1999; Zou *et al*., 1999; Jako *et al*., 2001; Lu and Hills, 2002). The *DGAT1* gene mutant *as11* slows DAG accumulation, which lowers the TAG/DAG ratio (Katavic *et al*., 1995; Hobbs *et al*., 1999; Routaboul *et al*., 1999; Zou *et al*., 1999; Jako *et al*., 2001). The seed oil content of *as11* is decreased by 25–35% relative to the WT, and the fatty acid composition is altered with an increased linolenic acid (C18:3) content (Katavic *et al*., 1995; Zou *et al*., 1999; Lu and Hills, 2002). Although the oil content in mature *as11* seeds differs from the WT, there is no significant difference between *as11* and WT in regard to morphology (Katavic *et al*., 1995; Zou *et al*., 1999; Lu and Hills, 2002), dry weight, fresh weight and protein levels (Katavic *et al*., 1995). However, *as11* seeds show delayed germination, which can be recovered by treatment with a high concentration of mannite (Routaboul *et al*., 1999; Lu and Hills, 2002). Additionally, the sucrose content in dry seeds of *as11* was higher than in the WT, and increased and then decreased after absorbing water and finally fell to the same level as that of the WT (Lu and Hills, 2002).

In recent decades, the majority of studies examining the *DGAT1* mutant *as11* have primarily focused on genetics, molecular biology, physiology and biochemistry (Katavic *et al*., 1995; Hobbs *et al*., 1999; Routaboul *et al*., 1999; Zou *et al*., 1999; Jako *et al*., 2001; Lu *et al*., 2003; Zhang *et al*., 2009; Xu *et al*., 2012). Studies on the total lipid and TAG content during pollen development in *as11* are rare. Our transcriptional analysis found that the expression levels of key genes in the TAG synthesis pathway were downregulated in mature pollen, and the amount of endoplasmic reticulum (which indirectly influences the lipidosome content in the tapetum) was different in the mutant, ultimately resulting in a decrease in the total lipid and TAG contents in the mature pollen and in changes in fatty acid composition and expression of genes related to lipid synthesis during pollen development.

## Methods

### Plant material

Seeds of *A. thaliana* ecotype Columbia and *DGAT1* mutant *as11* were sown onto damp nutrient soil and cold treated for at least 2 days at 4 °C. Then seedlings were placed into an incubator with illumination for 16 h and darkness for 8 h per 24-h cycle. Plants with normal growth and development were used for the experiments.

###  Thin section and histochemical staining

Flower buds at different developmental stages were harvested and processed according to a previously described method, with a few modifications (Zhang *et al*., 2002). Semithin sections (1 μm thick) were cut using a Leica RM2255 microtome, stained with PAS and Sudan Black B, and then observed and photographed under a Leica DMLB. Ultrathin sections (60–70 nm) were cut using a Leica UC6 ultramicrotome, stained with uranyl acetate and lead citrate, and observed and photographed under a Philips FEI-TECNAI 12 transmission electron microscope.

### Total lipid and TAG quantification and total fatty acid profiles

Mature anthers were harvested in glass tubes on dry ice and weighed after freeze drying. Total lipid was extracted using the chloroform/methanol method described in the Acyl-Lipid Metabolism Chapter of The Arabidopsis Book (2013). TAG was fractionated by TLC (Silica gel 60, Merck, USA) in developing solvent (hexane: diethylether: acetic acid = 70: 30: 1 (v/v/v)) and visualized by iodine vapor. Spots were scraped for following fatty acid methyl esters (FAME) with TAG standard (C15:0) to quantify the content of TAG.

FAME of TAG and anthers at the VM (vacuolate microspore), BM (binuclear pollen grain), SMD (second mitotic division) and MP (mature pollen grain) stages were produced by a direct acid-catalyzed transmethylation protocol presented in the Acyl-Lipid Metabolism Chapter of The Arabidopsis Book (2013), and analyzed by GC-MS (7890A-5975C, Agilent Technologies, USA). GC conditions were as follows—splitless mode injection, injector, and flame ionization detector (FID) temperature, 250 °C; oven temperature program—100 °C for 1 min, 15 °C min^–1^ to 175 °C for 12 min, then 2 °C min^–1^ to 245 °C, holding this temperature for 2 min.

### RNA isolation and qRT-PCR

Total RNA of anthers from the VM, BM, SMD and MP stages was extracted by RNA FAST200 (Fastagen, Shanghai, China). Reverse transcription was completed using a PrimeScript™ 1st Strand cDNA Synthesis Kit (Takara, Dalian, China). Expression of genes selected according to TAG synthesis (http://aralip.plantbiology.msu.edu/pathways/triacylglycerol_biosynthesis) was determined by 2× SYBR Premix Ex Taq with a CFX96 Real-Time PCR Detection System (Bio-Rad Laboratories, Inc., USA). *Arabidopsis thaliana Actin2* gene was used as the housekeeping gene. Three biological replicates were done for each gene. The primers are listed in Supporting Information—Table S1.

### Library preparation for transcriptome sequencing

Total mature anther RNA for each sample (3 biological replicates) was extracted using the TRIzol method (TIANGEN BIOTECH, Beijing) and treated with RNase-free DNase I (TaKaRa). RNA degradation and contamination was monitored on 1% agarose gels. RNA was quantified using an Agilent 2100 Bioanalyzer (Agilent Technologies, CA, USA), and its quality and integrity were assessed using a NanoDrop spectrophotometer (IMPLEN, CA, USA). A total of 1.5 μg RNA per sample was used as input material for the RNA sample preparations. Sequencing libraries were generated using the NEBNext^®^ Ultra™ RNA Library Prep Kit for Illumina® (NEB, USA) following the manufacturer’s recommendations, and index codes were added to attribute sequences to each sample. Briefly, mRNA was purified from total RNA using poly-T oligo-attached magnetic beads. Fragmentation was carried out using divalent cations under elevated temperature in NEBNext First Strand Synthesis Reaction Buffer (5×). First-strand cDNA was synthesized using random hexamer primers and M-MuLV Reverse Transcriptase (RNase H). Second-strand cDNA synthesis was subsequently performed using DNA Polymerase I and RNase H. Remaining overhangs were converted into blunt ends via exonuclease/polymerase activity. After adenylation of the 3ʹ ends of DNA fragments, NEBNext Adaptor with a hairpin loop structure was ligated to prepare for hybridization. In order to select cDNA fragments of preferentially 200–250 bp in length, the library fragments were purified with the AMPure XP system (Beckman Coulter, Beverly, USA). Then 3 μL USER Enzyme (NEB, USA) was used with size-selected, adaptor-ligated cDNA at 37 °C for 15 min followed by 5 min at 95 °C before PCR. Then PCR was performed with Phusion High-Fidelity DNA polymerase, universal PCR primers and Index (X) Primer. Finally, PCR products were purified (AMPure XP system) and library quality was assessed on the Agilent Bioanalyzer 2100 system. The library preparations were sequenced on an Illumina Hiseq 4000 platform by the Beijing Allwegene Technology Company Limited (Beijing, China) and paired-end 150 bp reads were generated.

### Quality control

Raw data (raw reads) of fastq format were firstly processed through in-house perl scripts. In this step, clean data (clean reads) were obtained by removing reads containing adapter, reads containing ploy-N and low-quality reads from raw data. At the same time, Q20, Q30, GC-content and sequence duplication level of the clean data were calculated. All the downstream analyses were based on clean data with high quality.

### Quantification of gene expression levels

HTSeq v 0.5.4 p3 was used to count the read numbers mapped to each gene. Gene expression levels were estimated by fragments per kilobase of transcript per million fragments mapped (FPKM).

### 2.8 Differential expression analysis

Differential expression analysis of two groups was performed using the DESeq R package (1.10.1). DESeq provides statistical routines for determining differential expression in digital gene expression data using a model based on the negative binomial distribution. The resulting P values were adjusted using the Benjamini and Hochberg’s approach for controlling the false discovery rate. Genes with an adjusted P-value < 0.05 found by DESeq were classified as differentially expressed.

### 2.9 GO enrichment analysis

Gene Ontology (GO) enrichment analysis of the differentially expressed genes (DEGs) was implemented by the GOseq R packages based Wallenius non-central hyper-geometric distribution (Young *et al*., 2010), which can adjust for gene length bias in DEGs.

### 2.10 KEGG pathway enrichment analysis

KEGG (Kanehisa *et al*., 2008) is a database resource for understanding high-level functions and utilities of biological systems, such as the cell, the organism and the ecosystem, based on molecular-level information, especially large-scale molecular datasets generated by genome sequencing and other high-throughput experimental technologies (http://www.genome.jp/kegg/). We used KOBAS (Mao *et al*., 2005) software to test the statistical enrichment of differentially expressed genes in KEGG pathways.

### 2.11 Data analysis

Figures were plotted by Origin2018 (Microcal Software Inc., Northampton, MA). All histograms were processed with Excel (Microsoft, Seattle, WA). The data were statistically analyzed using SPSS version 20.0 (SPSS Inc., Chicago, IL). One-way ANOVA (Duncan’s multiple range test) was performed and differences were considered significant at p < 0.05.

## 3 Results

### 3.1 Transcriptome analysis revealed candidate genes involved in TAG biosynthesis

**Fig. 1.**
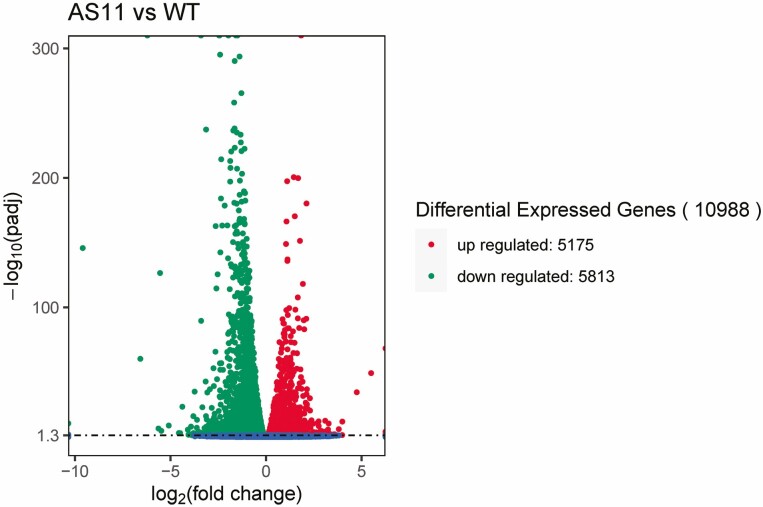
Differentially expressed genes in WT and *as11*. Volcano plot of DEGs between WT and *as11.* Each dot in the volcano plot represents a gene; the abscissa represents the logarithm of the difference multiple of gene expression between the WT and *as11* lines, and the ordinate represents the negative logarithm of statistically significant changes in gene expression. The larger the absolute value of the abscissa, the greater the multiple difference of expression quantity between the two lines; the larger the vertical coordinate value, the more significant the differential expression level, and the more reliable the selected DEGs. The green dots represent downregulated DEGs, the red dots represent upregulated DEGs, and the blue dots represent no DEGs. In comparing the gene expression in the WT and mutant, 10988 DEGs were consistently expressed in both the WT and mutant, 5175 were upregulated and 5813 were downregulated in the mutant.

**Fig. 2.**
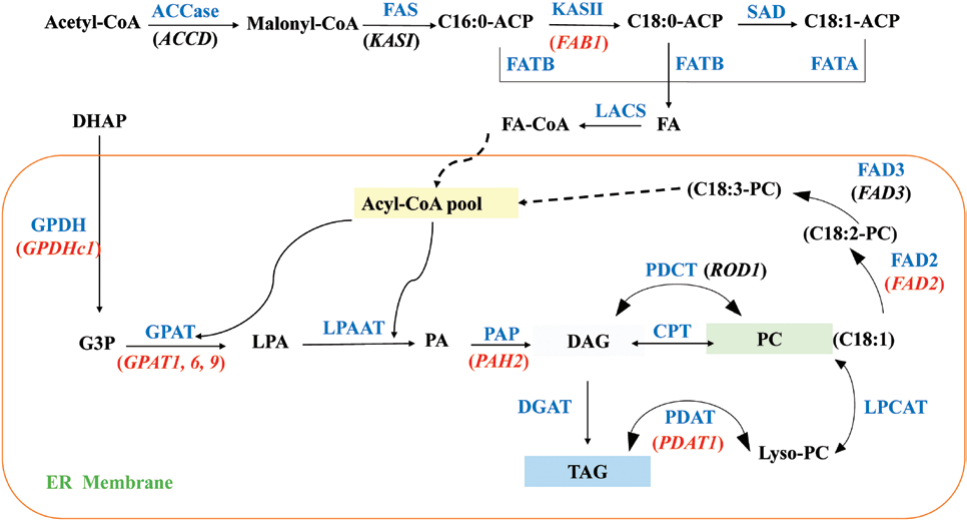
Overview of the TAG synthesis pathways in *Arabidopsis*. The genes marked in red were chosen for the expression profile analysis, and were downregulated in *as11* according to the RNA-seq data. DHAP, dihydroxyacetone phosphate; GPDH, glycerol-3-phosphate dehydrogenase; G3P, glycerol-3-phosphate; GPAT, glycerol-3-phosphate acyltransferase; LPA, lysophosphatidic acid; LPAAT, lysophosphatidic acid acyltransferase; PA, phosphatidic acid; PAP, phosphatidic acid phosphatase; DAG, diacylglycerol; DGAT, diacylglycerol acyltransferase; CPT, CDP-choline: diacylglycerol cholinephosphotransferase; PDCT, phosphatidylcholine: diacylglycerol cholinephosphotransferase; PLC, phospholipase C; PLD, phospholipase D. PDAT, phospholipid:diacylglycerol acyltransferase; TAG, triacylglycerol.

Using the Illumina HiSeq4000 sequencing platform and PE150 sequencing strategy, 141,560,394 raw read pairings were measured and 139,274,010 clean read pairings were obtained after quality control. A total of 10,988 differential genes were identified, of which 5,175 were upregulated and 5,813 were downregulated in *as11* (Fig. 1). GO enrichment analysis of differentially expressed genes resulted in 10,562 genes being annotated into 30 subclasses of two GO classes: biological processes and cellular components. A total of 6573 DEGs were identified as being involved in cellular processes in the biological process domain, and 7910 and 7920 DEGs were identified as being involved in cell parts and cells in the cellular component morphogenesis domain respectively. KEGG enrichment analysis of differentially expressed genes identified 2109 DEGs. In *as11*, 2105 DEGs were upregulated and 704 DEGs were downregulated. They were mainly enriched in metabolic pathways (383 upregulated, 460 downregulated) such as biosynthesis of secondary metabolism, carbon metabolism, biosynthesis of amino acids, fatty acid metabolism and fatty acid degradation (fatty acid metabolism) **[**see Supporting Information—Fig. S1]. Forty-eight of the 69 related genes identified in the fatty acid metabolism pathway were differentially expressed, and 41 of the 60 genes identified in the glycerolipid metabolism pathway were differentially expressed. Among the 43 genes identified in the fatty acid biosynthesis pathway, 28 were differentially expressed. According to the TAG synthesis pathway (Fig. 2), 8 key genes with changes in expression were identified (Table 1), and subsequent studies of these were carried out.

**Table 1:** Genes with changes of glycerolipid etabolism in transcriptomics.

DEGs	EC No.	Gene Description	log2F	PValue	Express differences
AT2G41540	1.1.1.8	Glycerol-3-phosphate dehydrogenase [NAD(+)] GPDHC1, cytosolic (***GPDHc1***)	-0.4291	2.86E-23	Down
AT1G06520	2.3.1.15	Glycerol-3-phosphate acyltransferase 1 (***GPAT1***)	-0.54064	2.24E-25	Down
AT2G38110	2.3.1.15	Glycerol-3-phosphate 2-O-acyltransferase 6 (***GPAT6***)	-0.98036	1.45E-137	Down
AT5G60620	2.3.1.15	***GPAT9***	-0.49674	1.05E-11	Down
AT5G42870	3.1.3.4	Phosphatidate phosphatase PAH2 (***PAH2***)	-0.31911	3.12E-07	Down
AT5G13640	2.3.1.158	Phospholipid:diacylglycerol acyltransferase 1 (***PDAT1***)	-0.49734	8.15E-18	Down
AT3G12120	1.14.19.6 1.14.19.22	***FAD2***	-0.20497	1.72E-08	Down
AT1G74960	2.3.1.179	3-oxoacyl-[acyl-carrier-protein] synthase II, chloroplastic (***FAB1***)	-0.23545	5.45E-06	Down
AT5G24300	2.4.1.21	Starch synthase, chloroplastic/amyloplastic (***SS1***)	0.23372	0.0021172	Up
AT3G01180	2.4.1.21	Starch synthase, chloroplastic/amyloplastic (***SS2***)	0.19539	0.0029552	Up

### 3.2 The lower expression of genes related to lipid synthesis and content of total lipid and TAG in mature *as11* pollen

**Fig. 3.**
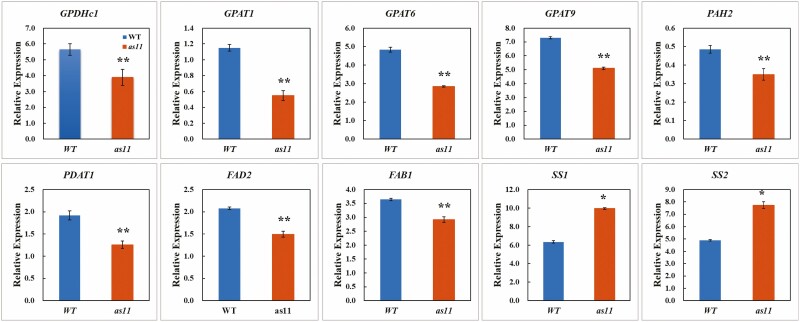
The relative expression of genes in the TAG synthesis pathway in the mature pollen grains. Significant differences are indicated by *P < 0.05 and **P < 0.01. Bars show the standard error, except when it was too small to be visible.

Based on transcriptome data, we next tested the expression of lipid synthesis related genes of wild type and mutant *as11* in mature pollen grains. Our results indicated that at the mature pollen grain stage, the expression of *GPAT* (*GPAT1, GPAT6,* and *GPAT9*) and *PDAT1*, which are involved in seed TAG synthesis, was downregulated in *as11* (Fig. 3). The *GPDHc1* gene, which regulates the transition from glycometabolism to lipid metabolism, was expressed at a higher level in the WT (Fig. 3). The expression analysis of the key genes related to fatty acid synthesis revealed that the expression of *FAB1*, which catalyzes the conversion of C16:0-ACP to C18:0-ACP, and the key enzyme gene *FAD2*, which can catalyze the production of linoleic acid (C18:2) was distinctly upregulated in the WT (Fig. 3). However, it is interesting that the expression of starch synthetase genes, particularly the *SS1* and *SS2* genes that are primarily responsible for the regulation of amylopectin synthesis, was higher in *as11* (Fig. 3).

**Fig. 4.**
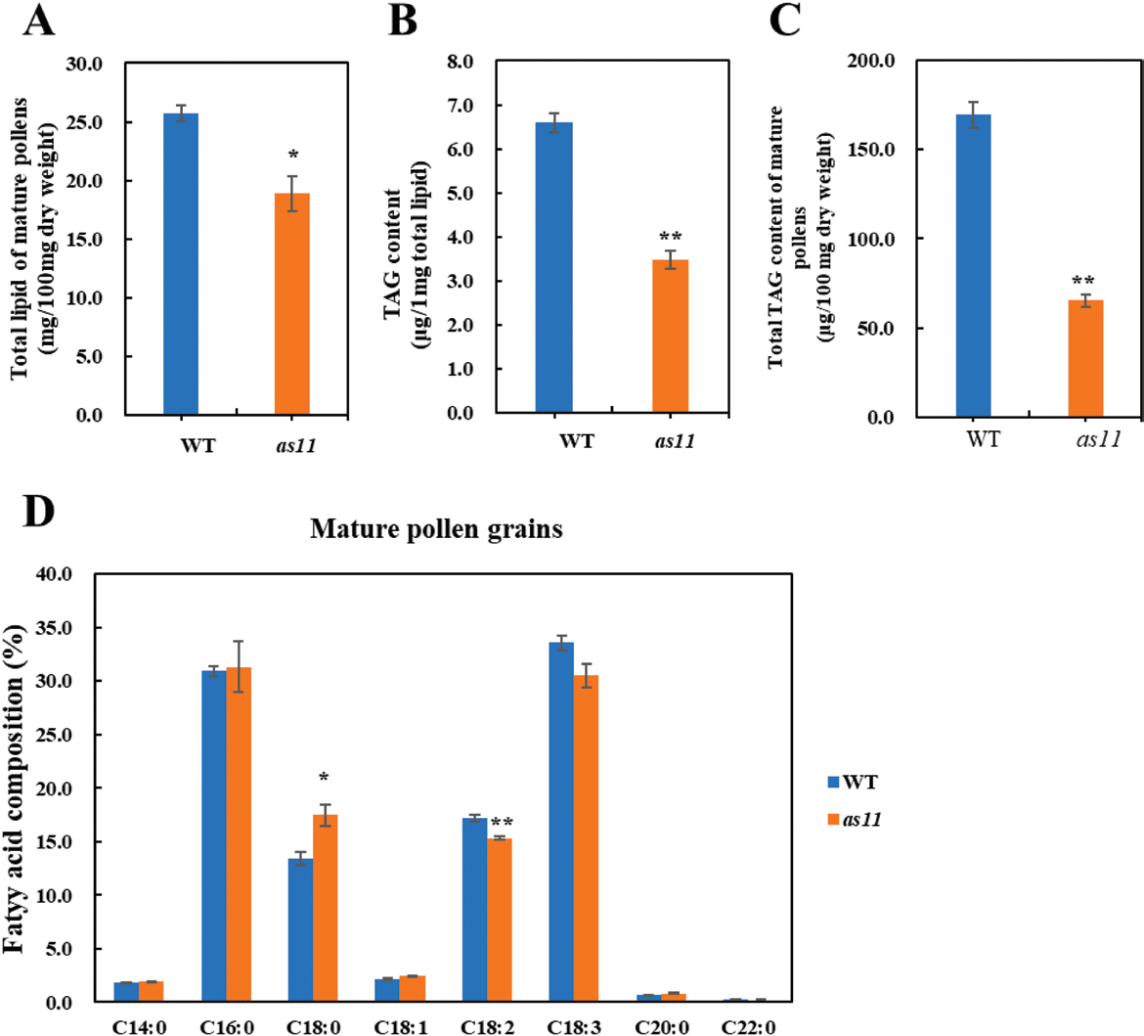
Comparison of total lipid, TAG levels and relative content of total fatty acid levels between anthers of the WT and *as11*. A. The content of total lipid. Based on 100 mg freeze-dried mature anthers, the content of total fat in WT mature anthers was 25.71 mg, and that of *as11* was 18.88 mg, 26.57% lower than that of the WT. B. The TAG of total lipid. 1 mg of the total lipid was separated by TLC, and GC-MS was used to calculate the TAG content: 6.60 μg/mg (TAG/total lipid) in WT mature anthers and 3.48 μg/mg in *as11* anthers. Compared with the WT, the content was 47.21% lower in the mutant. C. The total TAG in the anthers of the WT and *as11*. The content of TAG in the WT was 169.60 μg per 100 mg of freeze-dried mature anthers, while the content of TAG in *as11* was 65.32 μg, a decrease of 61.48% compared with that in the WT. D. The relative content of total fatty acid levels at the mature pollen grain stage. Significant differences are indicated by *P < 0.05 and **P < 0.01. Three biological replicates were performed.

Based on the lower expression of lipid synthesis related genes in *as11*, we further determined the content of total lipid and TAG in mature pollen grains of the WT and *as11*. The total lipid in mature pollen grains of *as11* was 26% less than that in the WT (Fig. 4A). The TAG content of the total lipid was also significantly lower in *as11* (Fig. 4B). As a result, the total content of TAG in mature *as11* pollen grains was distinctly lower than that in the WT (Fig. 4C). We next measured the fatty acid composition of the mature WT and *as11* pollen grains, and found no significant difference in content (Fig. 4D). Palmitic acid (C16:0) and linolenic acid (C18:3) were the main fatty acids in mature pollen in both the WT and *as11*. The content of linoleic acid (C18:2) and linolenic acid (C18:3) was a little higher in the WT. The stearic acid (C18:0) content followed the inverse pattern.

We also measured the fatty acid composition of wild type and mutant *as11* pollen grains at the VM (vacuolate microspore), BN (binuclear pollen grain), and SMD (second mitotic division) stages, and found that the content of linolenic acid (C18:3) was much higher in *as11* than in the WT at the SMD stage, presenting a ‘trade-off’ with linoleic acid (C18:2), palmitic acid (C16:0) and stearic acid (C18:0). The relative content of fatty acids at the VM stage was used as the standard to obtain the relative ratio of palmitic acid (C16:0), stearic acid (C18:0) and linolenic acid (C18:3) at four developmental stages. We found that at the SMD stage, the ratios of palmitic acid (C16:0) and stearic acid (C18:0) were lower in *as11* than in the WT (Fig. S3). Accordingly, the ratio of linolenic acid (C18:3) was significantly higher in *as11* than in the WT at the same stage. When the grains were mature, the ratio of the three fatty acids in both recovered the same between WT and as11.

### 3.3 The pollen ultrastructure changed during *as11* pollen development

Based on the developmental characteristics of the tapetum and pollen grains in the WT (Col ecotype) and according to the 13 growth stages of the entire developmental period in the wild-type *Arabidopsis thaliana* ecotype Wassilewskija (Owen and Makaroff, 1995), we divided the mature pollen grain stages of *Arabidopsis thaliana* ecotype Columbia into mature I and mature II stages, giving a total of 14 growth stages. The growth stages of mutant *as11* were divided on the basis of pollen development.

**Fig. 5.**
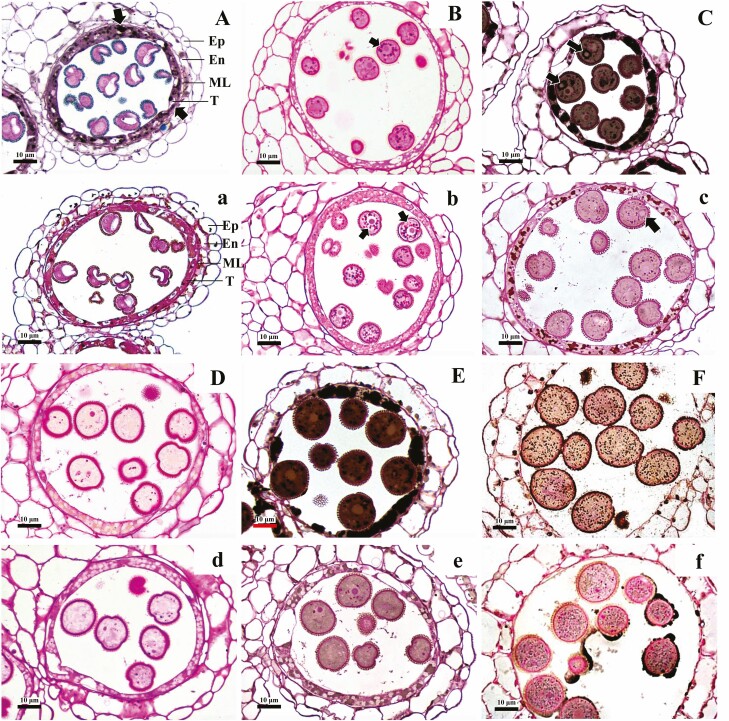
Anther cross sections of WT (A–F) and mutant *as11* (a–f) with PAS and Sudan Black B staining at different developmental stages. A, a: vacuolate microspore stage stained with PAS and Sudan Black B, A. Lipid was accumulated in the tapetum (arrows). B, b, C, c: binuclear pollen grain II stage (B and b were stained with PAS only, C and c were stained with PAS and Sudan Black B). B, b: starch grains were accumulated around the vegetative nucleus (arrows). C, c: lipid droplets deposited around the generative nucleus (arrows). D, d, E, e: second mitosis division I (D and d were stained with PAS only, E and e were stained with PAS and Sudan Black B), a few starch grains in the pollen grains; E, e: lipid droplets were accumulated in the tapetum cells and pollen grains, with fewer in the mutants (e). F, f: mature pollen grain II stage stained with PAS and Sudan Black; F, middle layer and tapetum had disappeared, and lipid droplets were accumulated in pollen grains, with fewer in the mutants (f). Bar = 10 μm. Ep, epidermis; En, endothecium; ML, middle layer; T, tapetum.

**Fig. 6.**
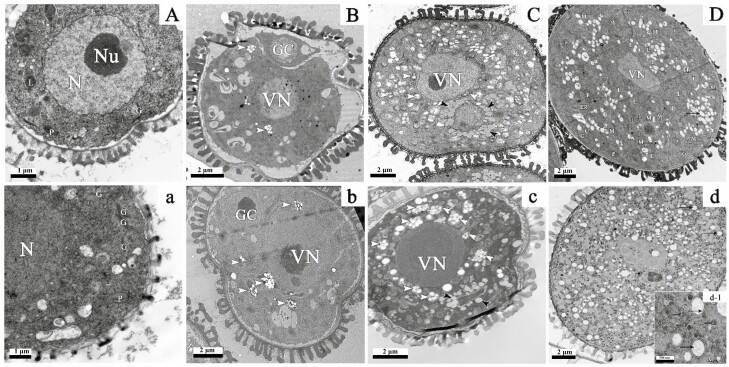
The ultrastructure of pollen grains in the WT (A–D) and *as11* (a–d) at different developmental stages. A, a: released microspore II stage; A: small lipid droplets occurred in microspores; a: the content of Golgi bodies was higher in microspores compared with WT. B, b: bicellular pollen grain I stage; B: vegetative nucleus was surrounded by plastids with starch grains (arrowheads); b: plastids with starch grains distributed around vegetative nucleus (arrowheads). C, c: binuclear pollen II stage; C: starch grains were observed around the vegetative nucleus (white arrowheads), and lipid droplets were deposited at the surface of the generative nucleus (black arrowheads); c: starch grains increased (white arrowheads) and lipid droplets were present at the surface of the generative nucleus (black arrowheads). D, d: mature pollen grain II stage (d-1 is partial enlarged view of d); D: lipid droplets and electron-transparent vesicles (black arrows) were abundant; d: a small number of lipid droplets were stored in mature pollen, which contained abundant electron-dense vesicles (d-1 white arrows) and electron-transparent (d and d-1 black arrows) covered with endoplasmic reticulum. N, nucleus; Nu, nucleolus; ER, endoplasmic reticulum; P, plastid; M, mitochondrion; G, Golgi bodies; L, lipid droplet; GC, generative cell; VN, vegetative nucleus.

Histochemical staining results indicated that there was no significant difference in the accumulation and distribution of nutrients during pollen development in either the WT or *as11* from the pre-meiosis stage to the released microspore stage (Fig. S2). At the vacuolate microspore stage, black granular lipid could be observed only in the tapetum of the WT (Fig. 5A). At the binuclear pollen grain II stage, starch grains surrounded the vegetative nucleus in both the WT and *as11* according to PAS staining (Fig. 5B and b); however, the mutant exhibited more starch accumulation. After counterstaining with Sudan Black B, there was obvious lipid accumulation in both the tapetum and the pollen grains of the WT and *as11*, and lipid droplets within the pollen grains were predominantly distributed around the generative nucleus (Fig. 5C and c). Furthermore, the content of lipid droplets within WT pollen was significantly higher than that in *as11*. When pollen grains developed to the second mitosis I stage, the number of starch grains within the pollen grains decreased significantly (Fig. 5D and d). Tapetal cells began to degrade, and lipid accumulation within the pollen grains continued to increase, with more lipid droplets in the WT (Fig. 5E and e). At the mature stage WT pollen grains were rich in lipid droplets (Fig. 5F), and the lipid droplet content was significantly higher (Fig. 5f). Both mature WT and *as11* pollen only contained a small amount of starch grains (Fig. 6F and f).

To explore the results at the cytological level, the ultrastructure of both the WT and *as11* was observed at different developmental stages. At the released microspore II stage, the microspores had a centrally located nucleus that was surrounded by a small amount of endoplasmic reticulum, and these microspores began to secrete substances to form the pollen intine (Fig. 6A and a). By this stage, small lipid droplets had already begun to form in the WT (Fig. 6A), while more Golgi bodies were present in *as11* (Fig. 6a). At the binuclear pollen grain I stage, a lenticular-shaped generative cell produced by an asymmetric mitosis was located on one side of the pollen grain and surrounded by a continuous intine. Several plastids with starch grains were present around the vegetative nuclei of both WT and *as11* plants (Fig. 6B and b). For further development to the binuclear pollen grain II stage, generative cells were free in the cytoplast of the vegetative cells, and these cells contained abundant vesicles and organelles such as mitochondria, plastids, and others. Lipid droplets precipitated at the surface of the generative nucleus, and plastids containing starch grains were distributed around the vegetative nuclei in both the WT and *as11* (Fig. 6C and c). Compared to the WT, the content of lipid droplets was reduced but plastids that were rich in starch grains were more numerous in mutant *as11* at this stage. When pollen grains matured, the cytoplasm of WT vegetative cells was rich in lipid droplets and endoplasmic reticulum and contained numerous electron-transparent vesicles (Fig. 6D). In contrast, lipid droplets and endoplasmic reticulum were lacking in *as11* pollen (Fig. 6d); however, electron-transparent vesicles that were covered with endoplasmic reticulum (Fig. 6d and d-1) and electron-dense vesicles (Fig. 6d-1) were both abundant in the cytoplasm.

### 3.4 The pollen ultrastructural changes were related to ultrastructural changes in the elaiosomes and the lipidosomes in the tapetum

Most of the nutrients in pollen grains come from the tapetum, so we also observed and compared the ultrastructure of associated organelles in the WT and mutant *as11* tapetal cells at different developmental stages, with particular focus on the developmental processes of the elaiosomes and the lipidosomes.

**Fig. 7.**
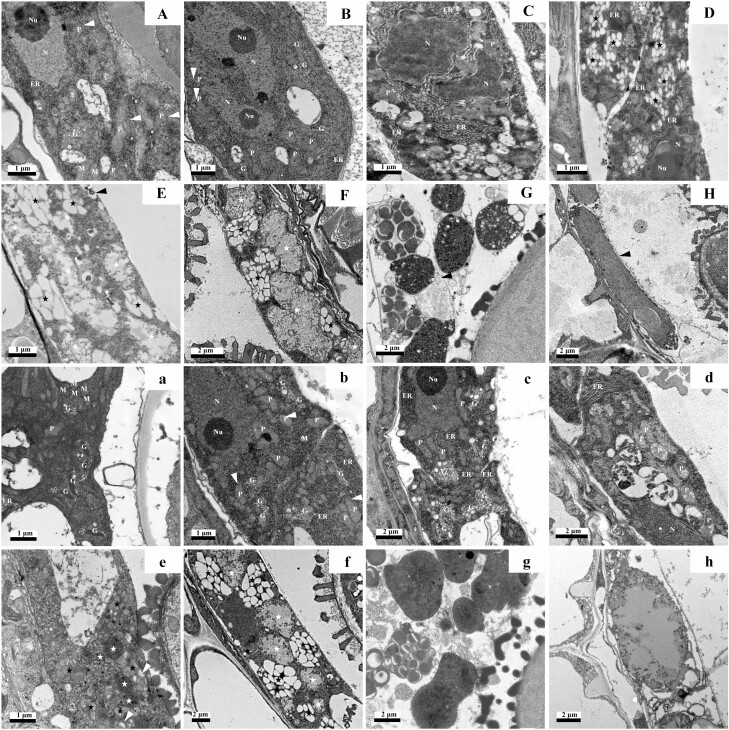
The ultrastructure of the tapetum in the WT and *as11* at different developmental stages. A, a: Post-meiosis, pre-cytokinesis stage. A: A few osmiophilic plastoglobules (white arrowheads) were visible in plastids. A: There were no plastoglobules in the cells. B, b: Tetrad stage. B: Osmiophilic and non-osmiophilic plastoglobules (black arrowheads) were accumulated in plastids. B: Osmiophilic plastoglobules (white arrowheads) occurred in plastids. C, c: Released microspore I stage. C: The endoplasmic reticulum content was abundant. C: The Golgi bodies were abundant with rich secretory vesicles. D, d: Vacuolate microspore stage. D: Rectangular chip-shaped substances (arrows) were presented in plastoglobules. D: Plastids started to accumulate non-osmiophilic plastoglobules (white arrowheads). E, e: Vacuolate microspores. E: Elaiosomes (black asterisks) became stable and lipidosomes (white asterisks) occurred. e: Rectangular chip-shaped substances (white arrows) were present in the plastoglobules. Elaiosomes (black asterisks) became stable and lipidosomes (white asterisks) occurred. F, f: Binuclear pollen grain I stage. Vesicle containing electron-dense materials fused with plasmalemma to release inclusions to anther locule (black arrows), and lipidosomes (white asterisks) coalesced with each other to form sheeting structures. G, g: nuclearpollen grain II stage. Elaiosomes (black asterisks) and lipidosomes (white asterisks) occupied most of the space in the tapetum. H, h: Second mitotic division I. H: Tapetal plasmalemmas broke down (white arrows) and freed lipidosomes and elaiosomes to deposit on the surface of the pollen extine. H: Tapetal plasmalemmas break down (white arrows) and freed lipidosomes and elaiosomes to anther locule. I, i: Second mitotic division II stage. I: Tapetal cells degraded completely, only leaving lamellate lipid structures fused by lipidosomes (black arrows). Tapetal cells degraded completely, only leaving lamellate lipid structures fused with lipidosomes (black arrows). N, nucleus; Nu, nucleolus; ER, endoplasmic reticulum; P, plastid; M, mitochondrion; G, Golgi bodies.

At the post-meiosis and cytokinesis stage in WT tapetum, a small number of Golgi bodies were present in the cytoplasm, but plastids were abundant, and osmiophilic deposits appeared to be internal (Fig. 7A). Osmiophilic deposits were not obvious within plastids in the mutant tapetal cells, and the Golgi bodies were present (Fig. 7a). At the tetrad stage, plastids in WT tapetum began to accumulate internal osmiophilic and electron-transparent deposits. The endoplasmic reticulum was abundant and primarily located between the nucleus and the cytomembrane. The Golgi bodies were highly developed (Fig. 7B). The tapetum of mutant *as11* exhibited an increase in the content of endoplasmic reticulum and these structures were also rich in Golgi bodies actively secreting vesicles. Osmiophilic deposits were also present in plastids (Fig. 7b). When microspores were released, in the WT tapetum numerous vesicles were stacked along the edge of plasmalemma, and the swollen endoplasmic reticulum was more obvious. The plastids were not significantly altered (Fig. 7C). In *as11*, the Golgi bodies were very abundant and were swollen with rich secretory vesicles. Osmiophilic droplets appeared to be increased within plastids (Fig. 7c). At the stage of vacuolate microspores in WT tapetum, the endoplasmic reticulum was still plentiful, and a large number of plastoglobules that exhibited a centered rectangular chip structure began to accumulate in plastids. Additionally, numerous expanded Golgi bodies and exuberant secretory vesicles developed into a relatively stable elaiosomes. New lipid-like structures called lipidosomes also became visible, which was surrounded by extensive endoplasmic reticulum (Fig. 7D). At the vacuolate microspore stage in *as11* tapetum, vesicles harboring electron-dense materials increased in number, and plastoglobules began to accumulate within plastids. At this stage, lipidosomes were not observed in mutant tapetal cells (Fig. 7d). At the binuclear pollen grain I stage, the plastoglobules formed a centered rectangular chip structure in plastids that subsequently became relatively stable elaiosomes. Additionally, a small number of lipidosomes appeared (Fig. 7e). As pollen grains continued to develop, the Golgi bodies were reduced and vesicles possessing electron-dense inclusions were still present in the WT tapetum. The elaiosomes remained stabilized, and the lipidosomes increased and fused with each other to form sheeting structures that were surrounded by plentiful endoplasmic reticulum (Fig. 7E). At the binuclear pollen grain II stage, the tapetal cells were primarily occupied by elaiosomess and lipidosomes, and the content of both structures reached its maximum (Fig. 7F). The development of mutant tapetal cells during the late binuclear pollen grain stage was not significantly different from that of the WT (Fig. 7f). The content of elaiosomes and liposomes, which occupied most of the volume in tapetal cells, also reached its peak at the binuclear pollen grain II stage; however, the lipidosome content was not as rich as that of the WT (Fig. 7f). At the second mitotic division I stage, the tapetal cells of both the WT and *as11* were degraded. The electron density of the lipidosomes increased, and the structure of the elaiosomes remained stable. The structures were both released into the anther loculus after the plasma membrane had degraded, and they were deposited as a covering for the extine of pollen grains to form the tryphine (Fig. 7G and g). At the second mitotic division II stage, the tapetum of the WT and *as11* continued to release degraded cellular inclusions, and ultimately, these cells became vacuolate sheeting lipid structures that were fused by the lipidosomes. It should be noted that the lipid structure in the mutant *as11* was less electron-dense and did not fill the entire tapetal residual structure compared to the observations in the WT (Fig. 7H and h).

**Fig. 8.**
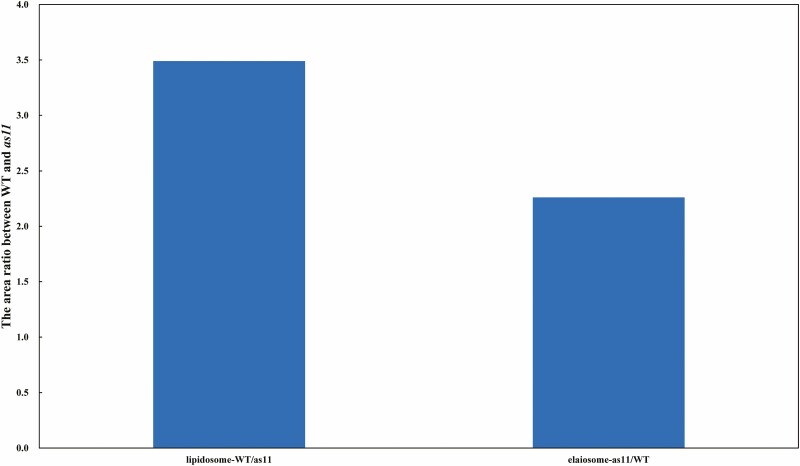
The area ratio of lipidosomes and elaisomes between WT and *as11*.

We randomly calculated the area of the lipidosomes and elaiosomes in 20 tapetal cells from the WT and mutant at the binuclear pollen grain stage, and found that the area of lipidosomes in WT tapetal cells was 3.49 times that of the mutant, while the area of the elaiosomes in mutant tapetal cells was 2.26 times that of the WT (Fig. 8).

## 4 Discussion

### 4.1 Fatty acid metabolism-related DEGs involved in *as11*

Transcriptome sequencing is now a routine experimental method for differential gene expression, quantitative gene expression, and transcriptional identification (Mao *et al*., 2021). Kim *et al*. (2016) analyzed and identified genes related to fatty acid biosynthesis in *Perilla frutescens* (L.) var frutescens by transcriptomic analysis. Studies on changes in the fatty acid metabolism in *Arabidopsis DGAT1* mutant *as11* seed have been relatively comprehensive, and the transcript profiling of developing WT and *as11* seed showed differences in the fatty acid metabolism gene expression (Zou *et al*., 1999; Aulakh and Durrett, 2019), but the genes related to fatty acid biosynthesis during pollen development of the mutant have not yet been clarified. Zhang *et al*. (2021) found fatty acid metabolism genes in *Ophiocordyceps sinensis* by transcriptomics. In the current study, a total of 10,988 differential genes were identified, of which 5,175 were upregulated and 5,813 were downregulated according to the transcriptome analysis (Fig. 1).

DGAT is an important enzyme catalyzing TAG synthesis in the Kennedy pathway, which is of great significance for TAG synthesis and accumulation (Zou *et al*., 1999; Lu and Hills, 2002; Lu *et al*., 2003). In addition to the Kennedy pathway, Dahlqvist *et al*. (2000) found that phospholipid:diacylglycerol acyltransferase (PDAT) in *Arabidopsis thaliana* can catalyze the synthesis of TAG. The *GPDHc1* gene expression product is glycerol-3-phosphate dehydrogenase with NAD dependence, which can directly catalyze the formation of dihydroxyacetone phosphate (DHAP) in the process of sugar metabolism to generate glycerol-3-phosphate (G3P), and then directly enter the Kennedy pathway to provide the glycerol framework for TAG synthesis. It plays an important role in the mutual conversion of sugars and lipids (Shen *et al*., 2006; To *et al*., 2012). The *FAB1* gene expression product β-Ketoacyl-ACP synthase II (KAS II) catalyzed the elongation of fatty acids from C16:0-ACP to C18:0-ACP (Hirano *et al*., 2001; Pidkowich *et al*., 2007).

Phosphatidylcholine:diacylglycerol choline phosphotransferase PDCT can transfer choline from C18:2/3-PC to C18:1-DAG and generate C18:2/3-DAG and C18:1-PC. The former can continue to synthesize C18:2/3-TAG. The latter, as a substrate for fatty acid dehydrogenase (FAD), continues to be desaturated to produce C18:2/3-PC (Lu *et al*., 2009). *PAH2* can encode phosphatidic acid phosphatase (PAP) and catalyze the production of diacylglycerol DAG (Eastmond *et al*., 2010). The DGAT activity of *as11* seeds decreased by 40–70% relative to the WT, which affected the amount of oil accumulation in seeds, especially the synthesis of TAG (Katavic *et al*., 1995; Zou *et al*., 1999), whereas the amount of α-linolenic acid (18:3) almost doubled (Zou *et al*., 1999; Jako *et al*., 2001; Lu and Hills, 2002), this being the main fatty acid for storing TAG in mature seeds (Zou *et al*., 1999). In the GO enrichment analysis of differentially expressed genes, 10,562 genes were annotated into 30 subclasses of two GO classes: biological processes and cellular components. Following a KEGG enrichment analysis of differentially expressed genes in fatty acid metabolism and fatty acid degradation and based on the results of functional enrichment analysis of DEGs, we selected fatty acids as the focus of research on the differences between WT and *as11*. The transcriptomic data of *as11* revealed that the expression levels of *GPGHC1*, *GPAT1*, *GPAT6*, *GPAT9*, *PDAT1*, *PAH2* and *FAB1* all decreased in *as11*. So, 7 genes related to TAG biosynthesis were selected for further analysis (Table 1).

### 4.2 Lipid accumulation and fatty acid composition change during pollen development in *as11*

Previous studies demonstrated that the content of oil and TAG in mature seeds of the Arabidopsis *DGAT1* gene mutant *as11* was distinctly lower than that in the WT (Katavic *et al*., 1995; Zou *et al*., 1999; Lu and Hills, 2002). The *DGAT1* gene can also reduce TAG accumulation in other nutritional organs of Arabidopsis (Slocombe *et al*., 2009). Additionally, the TAG content in mature seeds, roots, stems, leaves, and petals was decreased in tobacco after silencing of the *DGAT1* gene (Zhang *et al*., 2005). Antisense suppression of the *DGAT1* gene in *Brassica napus* not only decreased the oil content of mature seeds, but also reduced the seed yield and germination rate and resulted in highly abnormal development (Lock *et al*., 2009). Although plant pollen is very small, it is extremely abundant in nutrients such as lipid droplets and/or starch, which are needed to ensure that there is sufficient energy and material to promote the normal germination of pollen. Any abnormal accumulation of nutrients during pollen development may cause pollen sterility (Zhang *et al*., 2002, 2009; Nashilevitz *et al*., 2009; Li *et al*., 2012; Yang *et al*., 2017; Gao *et al*., 2019). As the main component of lipid stored in pollen grains, triacylglycerol (TAG) not only serves as an energy and carbon source, but it also participates in important metabolic processes such as membrane lipid remodeling and has great significance in regard to normal development and successful pollination (Yang and Benning, 2018). DGAT, the enzyme catalyzing the synthesis of TAG, is important for TAG synthesis and accumulation. It not only has a high content and activity in developing seeds, but also widely exists in different organs such as leaves, petals, fruits, and anthers (Hobbs *et al*., 1999; Zou *et al*., 1999; Lu and Hills, 2002; Lu *et al*., 2003). It was reported that during pollen development in *Arabidopsis thaliana* (Owen and Makaroff, 1995; Zajac, 1997; Zhang *et al*., 2002) and *Brassica napus* (Platt *et al*., 1998), lipid droplets first begin to accumulate at the binuclear pollen grain stage where they are mainly distributed around the generative nucleus, and these lipid droplets gradually increase as pollen develops until the pollen has matured. This phenomenon is not limited to cruciferous plants, but can also be observed in the pollen development of other plants such as *Lycium barbarum* (Xu *et al*., 2009) and *Pancratium maritimum* (Konyar, 2018), indicating that the binuclear pollen stage of pollen development is a critical period for the accumulation of important nutrients. The current study found that although *DGAT1* gene mutation did not cause pollen abortion, it seriously affected the lipid accumulation characteristics and content during pollen development. At the binuclear pollen grain II stage, although lipid droplets that accumulated in vegetative cells of mutant *as11* increased compared to the previous stage and were mostly distributed around the generative cell nucleus, the content was significantly less than that of the WT. In contrast, the number of plastids containing starch grains surrounding the vegetative nucleus was significantly higher in the mutant than in the WT (Fig. 5C and c; Fig. 6C and c). As the pollen developed, lipid continued to accumulate in pollen grains. Mature *as11* pollen was not as rich in lipid droplets as WT pollen (Fig. 5F and f; Fig. 6D and d). Furthermore there was less total lipid and TAG in mature *as11* pollen than in the WT (Fig. 4A and C). Previous studies revealed that seed oil in mutant *as11* was 25–35% lower than in the WT (Zou *et al*., 1999) and oil accumulated in seeds to approximately 70% of that in the WT (Xu *et al*., 2012). Our results revealed that the *DGAT1* gene also affected lipid and TAG synthesis and accumulation in pollen grains, and that mutating the *DGAT1* gene reduced the lipid and TAG content in pollen compared to that in seeds.

Fatty acids are important components of lipids. Heyl (1923) found that the pollen of ragweed contained saturated fatty acids such as lauric acid (C12:0), carmine acid (C14:0), and palmitic acid (C16:0) and unsaturated fatty acids such as oleic acid (C18:1) and linoleic acid (C18:2). Ching (1962) and Standifer (1966) detected fatty acids in pollen of five pine species and dandelion species respectively, and found that the contents of palmitic acid (C16:0), stearic acid (C18:0), oleic acid (C18:1), linoleic acid (C18:2) and linolenic acid (C18:3) were higher. Thus the fatty acids of 16-carbon palmitic acid (C16:0) and 18-carbon fatty acids are the main types in mature pollen. Zheng *et al*. (2003) measured fatty acids in mature pollen of WT and *GPAT1-1* mutants in *Arabidopsis thaliana*. They found that mutation of *GPAT1* affected the synthesis of linolenic acid (C18:3) in pollen. The fatty acids that accumulated in mature pollen of the WT and *as11* mutant were similar to those that accumulated in *Arabidopsis* seeds (Lu *et al*., 2009; Napier and Graham, 2010), and, consistent with the types of fatty acids in pollen measured by Zheng *et al*. (2003), palmitic acid (C16:0) and linolenic acid (C18:3) were the main fatty acids accumulated in the current study (Fig. 4D). However, we also measured the pollen fatty acid composition at the VM, BN, and SMD stages, and observed dynamic changes in fatty acid content during pollen development. The results showed that linolenic acid (C18:3) was the main fatty acid in pollen of the WT and *as11* during the second mitosis stage, with higher levels in the mutant (Fig. S3). Moreover, changes in the relative content ratio of linolenic acid in the anthers at each developmental stage (Fig. S3) indicated that the content and synthesis rate of linolenic acid (C18:3) in *as11* pollen grains were highest from the BN stage to the SMD stage. It has been reported that the *DGAT1* gene can affect the composition of fatty acids accumulated in seeds of *Arabidopsis thaliana*, in which the level of α-linolenic acid (C18:3) accumulation in mature seeds of as11 was nearly doubled compared with that of the WT (Katavic *et al*., 1995; Hobbs *et al*., 1999), being the main storage fatty acid for TAG in mature seeds (Zou *et al*., 1999). In conclusion, the *DGAT1* gene of *Arabidopsis thaliana* does not affect the type and content of fatty acids accumulated in pollen, but can affect the synthesis rate of linolenic acid. Therefore, we speculate that *DGAT1* affects the expression of genes related to linolenic acid (C18:3) synthesis.

### 4.3 Lipid accumulation and fatty acid composition may be related to the changes in TAG biosynthesis

In addition to the Kennedy pathway, TAG was found to be available through phospholipid:diacylglycerol acyltransferase (PDAT)-mediated catalysis of DAG using phospholipids in *Arabidopsis thaliana* (Dahlqvist *et al*., 2000). In *Arabidopsis* seeds, PDAT is not the primary determinant of TAG synthesis, as the low transcript levels of *PDAT1* in WT seeds were unchanged in *DGAT1* mutant seed (Aulakh and Durrett, 2019). However, after mutating the *DGAT1* gene, the expression of the *PDAT1* and *LPCAT2* genes was upregulated, and these pathways became the primary pathways for TAG synthesis and resulted in approximately 70% more oil accumulation in seeds than that in the WT (Xu *et al*., 2012). The failure to obtain a double mutant after crossing the *DGAT1* gene mutant and the *PDAT1* gene mutant indicated that the functions of *DGAT1* and *PDAT1* are complementary during pollen development (Zhang *et al*., 2009). However, our research found that the expression levels of *PDAT1* were higher in mature WT pollen grains. The expression of *GPAT9*, which is involved in seed TAG synthesis (Shockey *et al*., 2016), was also not clearly upregulated in *as11*. Zheng *et al*. (2003) found that the TAG content in flower buds of *GPAT1* gene mutants was decreased by approximately 10%, indicating that the *GPAT1* gene is involved in TAG synthesis in floral organs. The *GPAT6* gene, which is mainly expressed in the floral organs including the tapetum and microspores, could cause abnormal accumulation of nutrients in pollen and defects in pollen wall development by affecting the lipid metabolism of the tapetum, and this gene can also cause premature anther senility (Li *et al*., 2012). It was reported that the *GPAT1* and *GPAT6* genes exerted important effects on the normal development of Arabidopsis pollen (Zheng *et al*., 2003; Li *et al*., 2012). Additionally, the expression of the *PAH2* gene promotes the transformation of sugar to lipids and provides the glycerin skeleton for TAG synthesis, which catalyzes DAG synthesis and the *GPDHc1* gene (Shen *et al*., 2006; Eastmond *et al*., 2010; To *et al*., 2012). Recent research shows that the soybean *GPDHc1* gene is involved in lipid synthesis in the endoplasmic reticulum, and increases the content of C18:1 fatty acid in TAG by increasing the diacylglycerol (de novoDAG) produced by the G3P pathway (Zhao *et al*., 2021). So, we suggest that the high expression levels of *GPAT1* and *GPAT6* genes in the WT, in accordance with the trend for increased *GPDHc1* and *PAH2* expression in the same pathway of DAG synthesis, promotes TAG synthesis (Fig. 4) and the synthesis of endoplasmic reticulum in the tapetum. The synthesis of lipidosomes that are rich in TAG is closely related to that of the endoplasmic reticulum. Therefore, the high expression of *GPAT1* and *GPAT6* in the WT could promote the synthesis of endoplasmic reticulum in the tapetum. As a result, the obvious lipidosomes that were observed in the tapetal cells could provide raw materials for lipid synthesis in pollen.

*DGAT1* gene expression not only affects the content of oil in seeds, but can also alter the type of fatty acid accumulation. Compared to that in the WT, the accumulation of linolenic acid (C18:3) was twice as high in *as11* mature seeds (Katavic *et al*., 1995; Hobbs *et al*., 1999; Routaboul *et al*., 1999; Zou *et al*., 1999; Jako *et al*., 2001; Lu and Hills, 2002), and it is the main fatty acid stored in the TAG of mature seeds (Zou *et al*., 1999). Our study indicated that the main fatty acids in mature mutant *as11* pollen grains did not differ from that in the WT (Fig. 2D). However, during the SMD stage, linolenic acid (C18:3) became the main fatty acid in *as11*, based on the change trend of relative content of linolenic acid (C18:3) during development (Fig. S3). It was concluded that *DGAT1* increases the content of linolenic acid (C18:3) by regulating the expression of *FAD2* during pollen development. In *DGAT1* mutant seed, the transcript levels of *FAD2* encoding the ER-localized desaturases responsible for the synthesis of 18:2 and 18:3 were higher than that of the WT, and the desaturase genes that introduce double bonds in various plastidial glycerolipids were unaffected, like *FAB2* (Aulakh and Durrett, 2019). It was reported that the linolenic acid (C18:3) content was decreased in mutant *GPAT1-1* mature pollen grains and seeds (Zheng *et al*., 2003), and increased in mutant *GPAT9* mature seeds, compared to the WT (Shockey *et al*., 2016). Consistent with previous work, *GPAT1* was slightly upregulated in mutant seed (Zou *et al*., 1999; Aulakh and Durrett, 2019). Therefore, the high expression of *GPAT1* and slight upregulation of *GPAT9* gene expression in the WT may lead to a slight increase in linolenic acid (C18:3) content, and we speculated that this could be one of the reasons for the upregulation of *FAD2* gene expression. Furthermore, scanning electron microscopy indicated that there were few mature pollen grains in the mutant *GPAT1-1*, and this significantly reduced pollen fertility (Zheng *et al*., 2003). Based on this, the changes in pollen fatty acid composition may also exert effects on pollen fertility. The observed normal pollen morphology and self-pollination of *as11* (Katavic *et al*., 1995), however, indicate that to guarantee pollen sterility, the fatty acid composition of *as11* mature pollen grains should be significantly different. When the pollens were mature, the composition of the main fatty acids in *as11* tended to be similar to that in the WT (Fig. 5D). It was shown that in order to guarantee pollen sterility, *FAD2* expression decreased, which prevented the accumulation of linolenic acid, with the composition of fatty acids in the *as11* mature pollen gradually tending to be the same as the composition of the main fatty acids in WT pollen grains. Thus, expression of the *DGAT1* gene affects lipid accumulation and fatty acid composition during pollen development, but does not affect pollen fertility.

In conclusion, the *DGAT1* gene can affect the accumulation of TAG in pollen grains by affecting the expression levels of *GPDH*, *GPAT1*, *GPAT6*, *GPAT9* and *PAH2*, and affects the composition of fatty acids in pollen by affecting the expression levels of *FAB1* and *FAD2*.

### 4.4 The lower lipid content in *as11* may be indirectly related to the development of elaiosomes and lipidosomes in tapetal cells

As the innermost anther parietal cells, tapetal cells possess the function of absorbing and transporting polysaccharides or lipids to provide nutrients for the development and germination of pollen. Therefore, abnormal metabolism in tapetal cells will directly affect the development of microspores in the anther loculus and the macromolecular nutrient synthesis and accumulation that occurs in these cells. In addition to pollen grains, the tapetum also secretes lipids to nourish microspores during anther development (Zhu *et al*., 2011; Chen *et al*., 2013). Studies examining gene expression related to TAG synthesis using *in situ* labeling have shown that the anther is the second most active tissue besides the seed (Piffanelli *et al*., 1997, 1998; Murphy, 2001). In regard to the lipid cytological changes in the tapetum and pollen grains, Hernandez-Pinzón *et al*., (1999) isolated two organelles, the elaiosomes and the tapetosome, that were related to lipid synthesis by the tapetum of *Brassica napus*. The former is a type of oil-containing organelle that functions in the process of plant pollen development that involves sterol esters, TAG, and protein (Hernandez-Pinzón *et al*., 1999; Hsieh and Huang, 2004). The latter is a specific organelle in tapetal cells (Wu *et al*., 1997), and is rich in TAG and some proteins similar to seed oleosin. The tapetosome is closely related to the endoplasmic reticulum during anther development and is generally believed to be formed by rough endoplasmic reticulum budding (Hsieh and Huang, 2005). Similar structures have been observed in the tapetum of the *Arabidopsis thaliana* ecotype Columbia (Zhang *et al*., 2002; Suzuki *et al*., 2013), and these structures are called lipidosomes. The proteins in lipidosomes and sterol esters in elaiosomes are primarily used to synthesize the pollen coat. The specific function of TAG located within these structures is unclear, but it may be transported through the pollen coat by esterase or β-oxidation to the inner pollen for further metabolism and utilization (Zhang *et al*., 1994; Hernandez-Pinzón *et al*., 1999). In this study, we found that despite the fact that the lipidosomes and elaiosomes were both present in the tapetal cells of WT and *as11* mutants, there were differences in timing of occurrence and their characteristics.

First, plastids in the WT began to exhibit osmiophilic deposits at the post-meiosis and pre-cytokinesis stage (Fig. 7A), and reached a steady state during the vacuolate microspore stage when the lipidosomes formed (Fig. 7E). Plastids of mutant *as11* began to exhibit osmiophilic deposits at the tetrad stage (Fig. 7e), and reached a relatively steady state at the binuclear pollen grain I stage when the lipidosomes formed (Fig. 7f). Both organelles filled almost the entire tapetum in the WT and mutant at the binuclear pollen grain II stage (Fig. 7G and g), when the content reached its maximum. At the second mitosis stage, the tapetum was degraded and these structures were released into the anther loculus where they covered the surface of pollen grains. The area ratio of lipidosomes and elaiosomes showed that the area of lipidosomes was higher in the WT tapetal cells, while the area of elaiosomes was higher in mutant tapetal cells. Throughout the entire process of pollen development, the content of endoplasmic reticulum in WT tapetal cells was generally richer than that in *as11*, and the lipidosomes were primarily formed by rough endoplasmic reticulum budding (Platt *et al*., 1998; Ting *et al*., 1998; Hsieh and Huang, 2004, 2005). The *MAGL* gene (a homolog of *AtMAGL8*) which encodes the monoacylglycerol lipase (MAGL) in *Arabidopsis thaliana*, results in male sterility, and the lipidosome content in the tapetum was higher than that in the mutant (Gao *et al*., 2019). Based on this, the lipidosomes that were rich in TAG in the mutants appeared later and were less abundant than those observed in the WT. Zheng *et al*. (2003) examined the ultrastructure of *GPAT1* mutant anthers and found that the fusion of endoplasmic reticulum with plasma membranes occurred rarely and endoplasmic reticulum’s dilation was generally decreased. The ultrastructure of the *GPAT6* gene mutant anther also indicated that the content of the endoplasmic reticulum in tapetal cells was reduced and was difficult to detect in mature pollen grains (Li *et al*., 2012). The above results indicate that the relevant genes within the TAG synthesis pathway may also exert a certain impact on the development of the pollen. It has been reported that DGAT, an important enzyme encoded by the *DGAT1* gene, catalyzes the synthesis of TAG and is located on the endoplasmic reticulum (Routaboul *et al*., 1999; Zou *et al*., 1999; Jako *et al*., 2001; Kaup *et al*., 2002; Lu and Hills, 2002; Ramli *et al*., 2005). Thus, it is justifiable that in mutant *as11*, the decreased DGAT enzyme activity resulted in a lower content of endoplasmic reticulum in tapetal cells and pollen grains.

The elaiosomes contain only small amounts of TAG, and the reduction in DGAT activity should have no significant effect on its content. However, our study found that the content of elaiosomes in the mutant was higher than that in the WT. We speculated that the mutant may supplement TAG accumulation by increasing the number of elaiosomes. Compared to the WT, the content of Golgi bodies, which is closely related to the position of the elaiosomes, was relatively rich in mutant tapetum development. Studies have shown that the Golgi bodies are closely associated with the secretion of polysaccharides in plant cells (Buvat, 1989), as their membranes possess a variety of enzymes involved in polysaccharide synthesis. We considered that in the process of *as11* pollen development, due to the reduced TAG content, the *SS1* and *SS2* genes related to starch synthesis were clearly upregulated at the mature pollen grain stages to increase polysaccharide substances (Fig. 5B). As a result, the content of Golgi bodies that were associated with polysaccharide secretion also increased. Additionally, lipids are involved in the processing of secretory vesicles of the Golgi bodies (Kirchhausen, 2000; Shemesh *et al*., 2003; Weiss and Nilsson, 2003; Bethune *et al*., 2006). In particular, DAG is involved in the production of COP vesicles during the transport process facilitated by the Golgi-endoplasmic reticulum (Asp *et al*., 2009; Martinez-Alonso *et al.*, 2013), suggesting that DAG has a specific relationship with the transport of Golgi bodies and endoplasmic reticulum. The content of endoplasmic reticulum systems in tapetal cells of the mutants was less than that in the WT, and the reduction in TAG synthesis led to an increase in DAG accumulation. The Golgi body secreted vesicles increased instead of decreased endoplasmic reticulum vesicles.

Based on our findings, we concluded that the *DGAT1* gene regulates tapetum cell structure during pollen development. *DGAT1* indirectly regulates the activity characteristics of the lipidosomes and elaiosomes by affecting the contents of the endoplasmic reticulum and Golgi bodies and their secretory vesicle activity, and *DGAT1* ultimately affects the lipid accumulation in mature pollen grains.

## Supporting Information

The following additional information is available in the online version of this article –

Fig. S1. General bioinformatics analysis of all unigenes. A. GO classifications of the Venn diagram of annotated unigenes. B. Histogram of the KOG categories.

Fig. S2. Anther cross sections of WT (A–F) and mutant *as11* (a–f) with histochemical staining of PAS and Sudan Black at different developmental stages. A, a: premeiosis I stage, the anther wall composed of four layers, respectively epidermis, endothecium, middle layer and tapetum. The microspore mother cell has a large and distinct nucleus (arrow); B, b: premeiosis II stage, the microspore mother cells begin to deposit callose, the tapetum cells contract, and cytoplasm contains numerous vesicles (arrow). C, c: meiosis stage; C, the microspore mother cell was surrounded by callose, and the tapetum cells contracted with dense cytoplasm and a larger vesicle (arrow); c, the mother cell of the spore surrounded the callose, the tapetum cells contracted, with many small vesicles (arrow); D, d: Tetrad stage, after cytokinesis, the microspore mother cell formed four mononuclear microspores, which were enclosed and separated by callose (arrow). E, e: Released microspore I stage, callose wall was degraded and an irregular-shaped microspore was released into the pollen sac; only one nucleus was located in the microspore (arrow). F, f: Released microspore I stage, the microspore became more rounded and had thick walls. Ep, epidermis; En, endothecium; ML, middle layer; T, tapetum.

Fig. S3. Relative content of total fatty acids (A–C) and rate of relative content of three fatty acids (D–G) in WT and mutant *as11* at different developmental stages. VM, ring-vacuolate microspores; BN, binuclear pollen grains; SMD, second mitotic division; MP. Significant differences are indicated by *P < 0.05 and **P < 0.01. Three biological replicates were performed.

Table S1. The primer sequences for qRT-PCR.

plad012_suppl_Supplementary_Material

## Data Availability

The data underlying the study can be found in the manuscript and a supporting information file.
