# Supplementary material for: Changes in the content of pollen total lipid and TAG in Arabidopsis thaliana DGAT1 mutant as11
Source: AoB Plants. 2023 Mar 22;15(2):plad012. doi: 10.1093/aobpla/plad012 (PMC10100649; doi:10.1093/aobpla/plad012)
Supplement: plad012_suppl_Supplementary_Material [file plad012_suppl_supplementary_material.pdf]

A

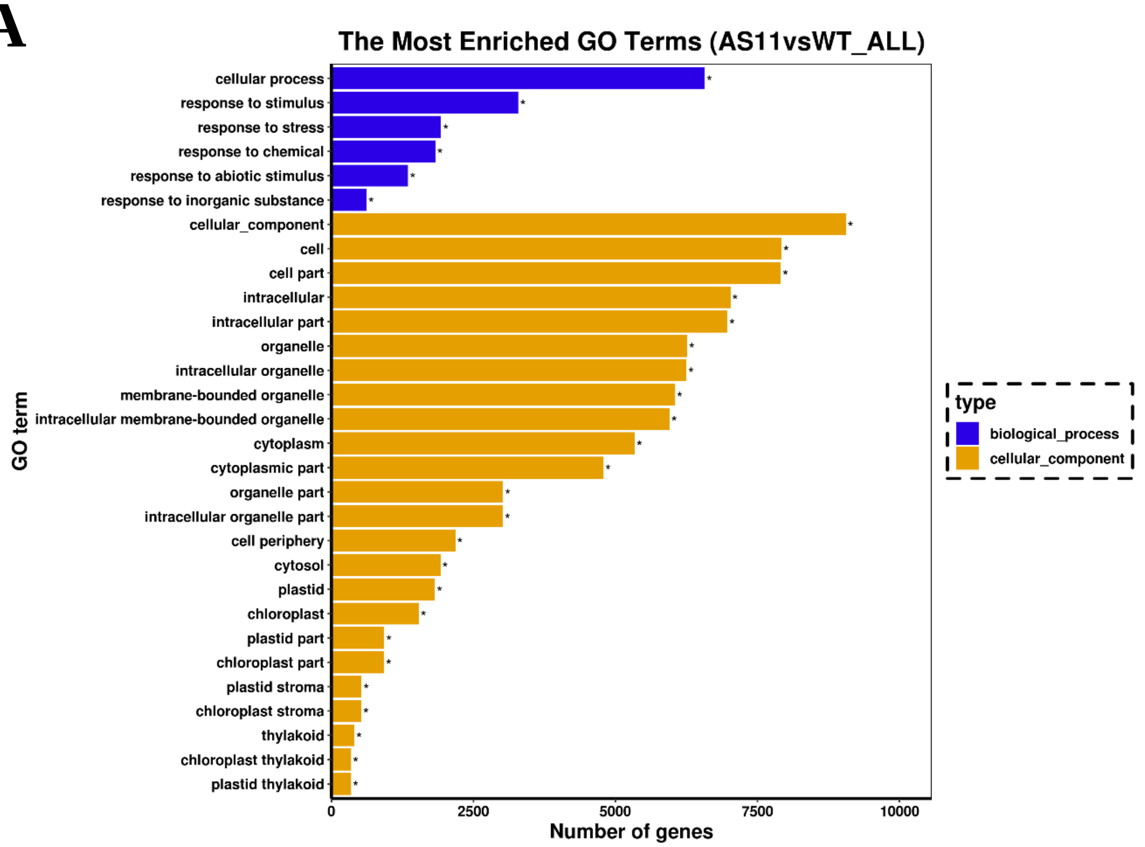

B

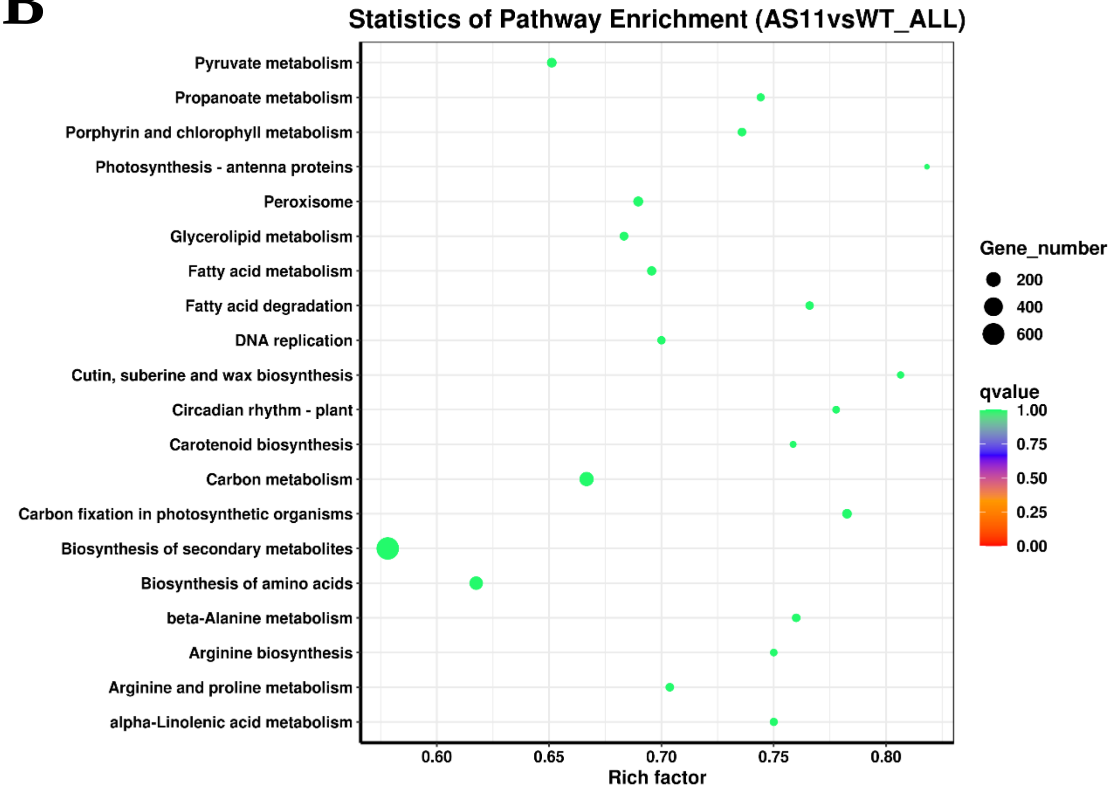

Fig. S1. General bioinformatics analysis of all unigenes.
A. Go classifications of the Venn diagram of annotated unigenes;
B. Histogram of the KOG categories.

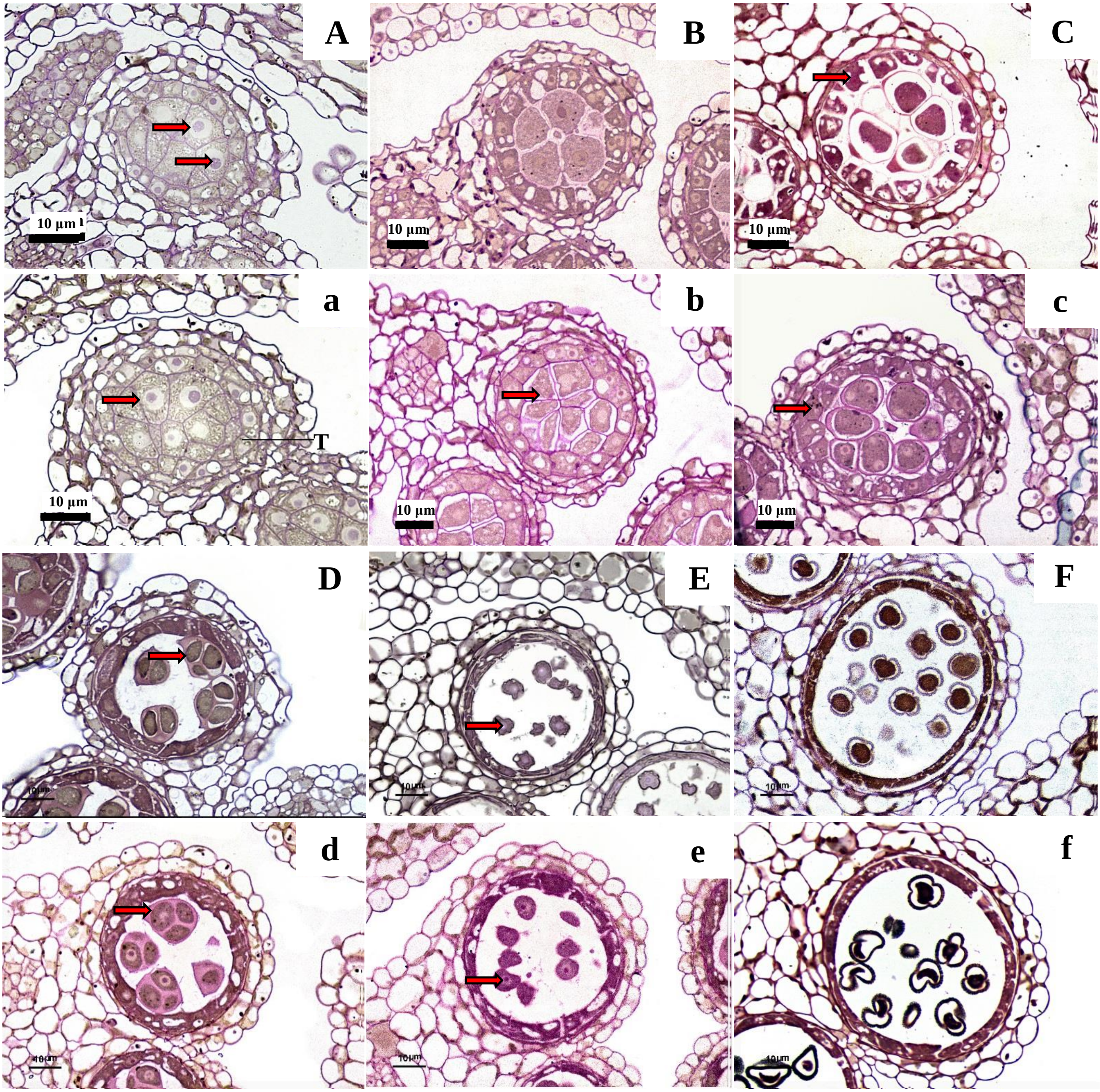

Fig. S2. Anther cross sections of WT (A–F) and mutant *as11* (a–f) with histochemical staining of PAS and Sudan Black at different developmental stages. A, a: premeiosis I stage, the anther wall composed of four layers, respectively epidermis, endothecium, middle layer and tapetum. The microspore mother cell has a large and distinct nucleus (arrow); B, b: premeiosis II stage, the microspore mother cells begin to deposit callose, the tapetum cells contract, and cytoplasm contains numerous vesicles (arrow). C, c: meiosis stage; C, the microspore mother cell was surrounded by callose, and the tapetum cells contracted with dense cytoplasm and a larger vesicle (arrow); c, the mother cell of the spore surrounds the callose, the tapetum cells contract, with many small vesicles (arrow); D, d: Tetrad stage, after cytokinesis, the microspore mother cell forms four mononuclear microspores, which are enclosed and separated by callose (arrow). E, e: Release microspore I stage, callose wall was degraded and an irregular-shaped microspore was released into the pollen sac; only one nucleus was located in the microspore (arrow). F, f: Release microspore I stage, the microspore becomes more rounded and has thick walls. Ep, epidermis; En, endothecium; ML, middle layer; T, tapetum.

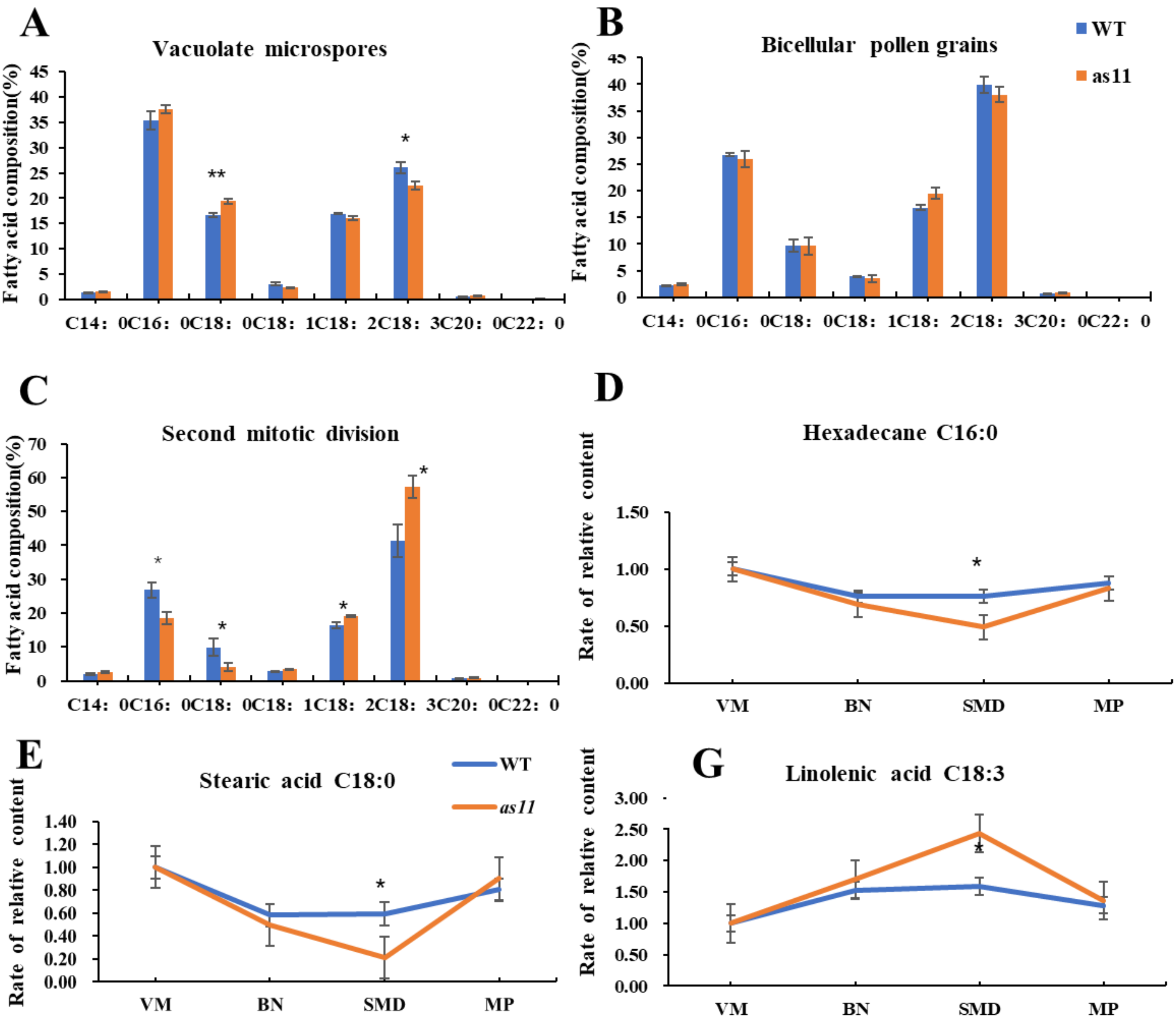

Fig. S3. Relative content of total fatty acid levels (A–C) and rate of relative content of three fatty acids (D–G) in WT and mutant *as11* at different developmental stages. VM, ring-vacuolate microspores; BN, binuclear pollen grains; SMD, second mitotic division; MP. Significant differences are indicated by *P < 0.05 and **P < 0.01. Three biological replicates were performed.

Table S1. The Primer Sequences of qRT-PCR

Gene	Forward（5'-3'）	Reserve（5'-3'）
FAB1	**CGTCTTCCTCTTACGCATCT**	**TGACGGCTATCTCCTCCACC**
GPDHc1	**CGCTACAAGCAAGTCGGTGT**	**GCAAGTTTCTCAGGCTCTTCTG**
GPAT1	**AGCGGGCTCAAGTGTTTAGA**	**AAAGCTTGACTTGCCTCCTG**
GPAT6	**TTGGTGGCCTAGCGTCTGATT**	**CGTACGTGGCACCATGTAACCT**
GPAT9	**GCAGGTCTATGTTGCCAACCA**	**AAGACCAACCCAACCAGGATG**
PAH2	**AACCAAGAACCGAGGCACTA**	**GGTCAGCTGAACCCAATGTT**
FAD2	**TAACGTTATCGCCCCTACGTCAGC**	**AATTGGTGGCGACGTAGTAGAAGCA**
PDAT1	**TTTCGAGGTGCTGTCAAAGG**	**CGCCATCATCTTAGGAGCAA**
SS1	**TGGGTCTTCACTCCCTTGTC**	**TCGGGTCATTCCTCTTCTCA**
SS2	**GCTCGCTGTCTTTTCGTTCT**	**ATCCTCTGGCTCATCATCGT**
PDAT1	**GTCGTACAACCGGTATTGTG**	**GAGCTGGTCTTTGAGGTTTC**
